# Effects of Varying the Color, Aroma, Bitter, and Sweet Levels of a Grapefruit-Like Model Beverage on the Sensory Properties and Liking of the Consumer

**DOI:** 10.3390/nu11020464

**Published:** 2019-02-22

**Authors:** Andries G. S. Gous, Valérie L. Almli, Vinet Coetzee, Henrietta L. de Kock

**Affiliations:** 1 Department of Consumer and Food Sciences, University of Pretoria, P O Box X20, Hatfield, Pretoria 0028, South Africa; gustavgous426@gmail.com; 2 Nofima AS, P O Box 210, 1431 Ås, Norway; valerie.lengard.almli@nofima.no; 3 Department of Genetics, University of Pretoria, P O Box X20, Hatfield, Pretoria 0028, South Africa; vinet.coetzee@up.ac.za

**Keywords:** grapefruit, sensory, consumer, bitter, naringin, sweet, aroma, color, hedonic

## Abstract

Color, aroma, sweet, and bitter tastes contribute to the sensory perception of grapefruit juice. Consumers differ about liking grapefruit. A reason is the bitter taste that characterize the fruit. The objective was to determine the effect of varying the color (red or yellow), aroma (two levels), bitterness (three levels), and sweetness (three levels) of a grapefruit-like model beverage, on consumers’ liking and perception of its sensory properties. The sensory profiles of thirty-six grapefruit-like beverages, created on the basis of a factorial design, has been described. Consumers rated their liking of color, aroma, and flavor of the twelve most diverse beverages. Bitter and sweet levels of the beverages had a significant effect on the flavor and aftertaste attributes. Aroma concentration had a significant effect on the majority of the sensory attributes. Color had a significant effect on perception of some of the aroma attributes, as well as the grapefruit’s flavor intensity. Consumers liked the red beverages more than the yellow ones, and those with low aroma over the high aroma intensity. Consumers preferred the low bitter/high sweet beverages. Pungent and grapefruit aroma were found to be negative drivers for liking of the aroma. Sweet and citrus flavors were found to be positive drivers and sour and bitter flavors were found to be negative drivers of flavor-preferences (or liking) of the tested beverages.

## 1. Introduction

The sensory properties of grapefruit (*Citrus X paradisi*) are distinctive characterizing components and play a key role in reasons why consumers choose or not choose to consume the fruit and its products, e.g., juice. Grapefruit is a rich source of vitamin C, and health-promoting citrus flavonoids and limonoids and has beneficial antioxidant and anti-inflammatory properties [1,2]. Its appearance, aroma, flavor, and mouthfeel properties contribute to the sensory perception of the fruit.

Consumers differ widely in opinions on liking or disliking grapefruit and part of this individual preference is attributed to liking or disliking of the bitter taste that characterizes the fruit. Excessive bitterness of the juice was considered to be an important problem in commercial grapefruit juice production [3]. Naringin and limonin are mainly responsible for the bitter taste commonly associated with grapefruit [4]. The consumption of fresh grapefruit, and grapefruit products has been declining [5] and plant breeders are working on ways to select for desirable sensory traits. A better understanding of the impact of the different sensory modalities contributing to the sensory perception of grapefruit products (e.g., juice) might assist product developers to optimize formulations and improve uptake of the products among consumers, thereby, maintaining or enhancing profitability for the role-players, along the grapefruit value chain.

Flavor perception is complex, due to the simultaneous stimulation of a number of senses. It is the result of processes that respond to sensory signals, from the activation of multiple sensory modalities, including smell (retronasal olfaction), mouthfeel (somatosensation), as well as taste (gustation), and to some extent also sight. When different senses are stimulated, concurrently, and perceptually interact with each other, the perceived flavor is the result of the cross-modal sensory interaction [6]. Cross-modal interactions can change the intensity and perceived character of individual tastes and aromas, and even the overall flavor [7].

The present study aimed broadly, to determine the relations between the stimulus components of a model beverage (formulated to be similar to grapefruit juice) and their effects on the perceived sensory properties and hedonic responses. We factorially combined, in the same acidified neutral base, each of three possible levels of bitter naringin (low, medium, and high) with each of three levels of sweet sucrose (low, medium, and high), two levels of grapefruit aroma (low and high) and two color variants (red and yellow). We hypothesized that perceived bitterness of the model grapefruit-like beverage will drive consumers dislike for the beverage but that bitterness perception will be a function of cross-modal color–taste, aroma–taste, and sweet–bitter taste interactions.

The color of the natural juice extracted from the grapefruit depends on the variety used and ranges from greenish-yellow to pale yellow, pink, and light red [8]. We hypothesized that a rose red grapefruit-like beverage would be perceived as sweeter than a pale yellow option. Previously, it was reported [9] that a red color decreased the perception of bitter taste intensity of a caffeine + water solution, with the yellow and green color having had no effect. The color of food and drinks impacts subsequent perception of taste, flavor, and overall sensory perception. It has been reported in several studies that the color of a solution greatly impacts the ability to identify its flavor and also affects the liking responses [7].

We hypothesized that a beverage with high, compared to a low grapefruit aroma, would suppress the bitter taste perception and enhance the taste of sweetness [10]. A new study [10] reported that lemon extract, sucrose, and citric acid, when presented separately and also together, affected the perception of sweet, sour, and citrus flavors. The aroma of the products can influence the perception of basic tastes and vice versa [11,12,13,14,15].

It is well-known that sucrose and other sweet tasting compounds can suppress bitterness. This is practically applied when bitter tasting coffee or tea is sweetened with sugar. Here the expectation was that sweetness would suppress bitterness but an enhancement effect on volatile aroma and flavor compounds was also expected. When sucrose was added to the fruit juices, not only were the perceived level of bitterness and sourness reduced and the sweet taste intensity increased, but the sweet aroma intensity rating also changed [16].

## 2. Materials and Methods

### 2.1. Preparation of the Grapefruit-Like Beverages

Thirty-six grapefruit-like beverages (Table 1) were manufactured, following a factorial design with deflavored, clarified, deionized, and acidified apple juice, as base, with an addition of naringin (three bitter levels), sucrose (three sweet levels), a grapefruit aroma compound mixture (two intensity levels) consisting of caryophyllene, citral, nootkatone, aldehyde C8 (octanal), aldehyde C9 (nonanal, aldehyde C10 (decanal), and two colorants (red or yellow). The addition of naringin was intended to reflect a low-level, in-between, and a high-level, based on the typical content in the grapefruit juice (218–340 mg/kg) [17]. The low level of sweetness was based on the industry minimum requirement for export purposes, with incrementally higher levels added to reflect medium and high sweetness. These aroma compound mixture and levels used were selected in consultation with a flavorant supplier. The typical grapefruit juice color was copied using artificial colorants. The red color was a 0.001% solution blend of 30% sunset yellow and 70% ponceau red. The yellow color consisted of 0.0125% quinoline yellow. Standard preparation and mixing procedures were used for all added stimuli to ensure uniformity. The grapefruit-like beverages were filled in 250 mL plastic bottles, with lids, for easy handling and uniformity, and were kept frozen at −18 °C, until use. The beverages were defrosted overnight at an ambient temperature and kept at 14 °C, until served. A summary of the physico-chemical characterization of the 36 grapefruit-like beverages is presented in the Supplementary Material (Table S1).

### 2.2. Descriptive Sensory Analysis

The sensory profiles of the beverages were described by a sixteen-member trained sensory panel with one to two years of descriptive sensory analysis experience. The specific training for attribute and methodology development for the evaluation of the beverages consisted of two sessions of 2 h each, using the generic descriptive analysis method [18]. A total of 21 attributes were generated to characterize the aroma, flavor, and aftertaste of the grapefruit-like beverages (Table 2). Beverage samples (±30 mL) were served at ±14 °C, in 125 ml polystyrene cups with plastic lids, and marked with randomly selected three-digit numbers. Samples were evaluated in duplicates, 12 beverages per 2 h session per day and a total of six sessions. The presentation order of samples per day for the different panelists followed a Williams Latin square design. Reference standards were available during training and evaluation sessions.

Panel performance was monitored to test reproducibility and consistency of the panel ratings using PanelCheck 1.3.2 (www.panelcheck.com; Nofima, Ås, Norway).

The attributes were evaluated on a structured horizontal line scale (10 cm) with descriptors at the scale ends ranging from ‘not intense’ (at the left end of the scale, 0 cm) to ‘very intense’ (at the right end of the scale, 10 cm). Data was captured using Compusense^®^ five release 4.6 software (Compusense Inc., Guelph, ON, Canada).

### 2.3. Consumer Evaluation

Ninety six young South African female consumers aged 18–24 years were recruited by trained fieldworkers. Each consumer completed an online screening survey and were invited to participate if in a self-reported good state of health, and if not limited by any food intolerance(s) and/or allergies. Participants were briefed and gave written consent before evaluating the beverages. Participants were requested not to eat, drink (except for water) or smoke for at least 1 h prior to the session.

The consumers (*n* = 90) evaluated liking of the color, aroma and flavor of the 12 most diverse beverages (selected on the basis of composition) (Table 1) using the Simplified Labeled Affective Magnitude (SLAM) scale [19], a 10 cm line scale labelled with descriptors ‘greatest imaginable dislike’ (at 0 cm), and ‘greatest imaginable like’ (at 10 cm). Sample preparation and presentation was the same as for the trained panel. The 12 samples were evaluated in one session and the order of presentation to different consumers followed a Williams design.

Data was captured using Compusense^®^ five release 4.6 software (Compusense Inc., Guelph, ON, Canada).

Ethical approval for this study was obtained from the Faculty of Natural and Agricultural Sciences Ethics Committee at the University of Pretoria (EC 130827-088).

### 2.4. Statistical Analysis

An analysis of variance (ANOVA) model fitted using PROC GLM in SAS v9.4 (SAS Institute Inc., Cary, NC, USA) was used to determine the main effects of the panelists, the bitter level, the sweet level, the aroma level, and the color type, together with the respective two-way interactions on the sensory attributes of the beverages. Tukey’s HSD test (*p* = 0.05) was used to compare beverages that differed in an attribute. Principal component analysis (PCA) using XLSTAT 2014 (Addinsoft, Paris, France) was applied to the correlation matrix of the sensory panel mean ratings, for all attributes of all grapefruit-like beverages.

Consumer liking of the color, aroma, and flavor of the 12 most diverse beverages was analyzed by a three-way ANOVA model, including the effects of color, aroma level, and tastants (bitter and sweet levels in three combination). Means were compared using Fisher’s least significant difference test at *p* < 0.05. Data were analyzed using GenStat^®^ (VSN International Ltd., Hertfordshire, UK). Consumer liking ratings (y) for color, aroma, and flavor of beverages were modeled as a function of the descriptive sensory attributes (x), using three separate partial least squares (PLS) regression models. Preliminary models were run with all sensory attributes and their squared terms. Variable importance (VIP), which measures how important a variable is in terms of modeling the liking attributes, was used to select a smaller number of linear and squared terms for the final model. The VIP values summarize the overall contribution of each X-variable to the PLS model, summed over all components, and weighted according to the Y variation, accounted for by each component. Only those linear terms with a VIP greater than 0.8, as well as the five squared terms with the highest contribution, were retained. The PLS models were used to determine the positive and negative drivers of color, aroma, and flavor liking, and also to predict consumer liking of the 24 samples that were profiled by the descriptive sensory panel, but not evaluated by the consumers. The SIMCA-P package (Umetrics, Umea, Sweden) was used for the PLS modeling.

## 3. Results

### 3.1. Descriptive Sensory Profiles of the Grapefruit-Like Beverages

Table 3 presents a summary of the main effects (color, aroma, bitter, and sweet) and two-way interaction ANOVA effects (provided in Supplementary Tables S2, S3 and S4) on sensory attributes of grapefruit-like beverages, as evaluated by the trained sensory panel. Means for each of the samples represent the average of duplicate ratings by 16 panelists. Color of the grapefruit-like beverages had a significant effect on perception of some aroma and flavor properties. The overall aroma and grapefruit, deteriorated/rotten, muddy/mouldy, fruity and sweet aroma, and grapefruit flavor of the red colored beverages were perceived as significantly (*p* < 0.05) more intense than the yellow colored beverages.

The level of aroma added had a significant effect on the majority of the sensory attributes, namely, overall aroma intensity and citrus, grapefruit, chemical, muddy/moldy, fruity, green/grassy, peely/peel oil, soapy, pungent, woody/spicy, and sweet aroma, with the lowest intensities perceived in the beverages with the low aroma level added. Aroma level had a significant effect on the bitter, astringent, and citrus flavor, and the bitter aftertaste perception, with the highest bitter and astringent flavor and bitter aftertaste being perceived in the beverages with a low aroma level and the highest citrus flavor being perceived in the beverages with a high aroma level.

Varying the naringin content (bitter level) of the beverages did not have any significant effect on any of the aroma attributes. It did, however, have a significant effect on the intensities of overall flavor and the astringent flavor, with the highest values observed for beverages with medium and high naringin concentrations. The naringin level had a significant effect on the intensities of sweet, sour, bitter, and grapefruit flavor, and the bitter aftertaste perception. The highest sweetness, but lowest sourness and grapefruit flavors were perceived in the beverages with low and medium naringin concentrations. Intensity of bitter flavor and bitter aftertaste followed the level of bitter compound addition.

Sweetness level contributed by sucrose had a significant effect on the perception of the many sensory properties of the grapefruit-like beverages. Significantly higher soapy aroma was perceived in the beverages with low and medium levels of sucrose, compared to a high sucrose addition. Sucrose level in the beverages had a significant effect on sour, sweet, bitter, astringent, and grapefruit flavor, and the bitter aftertaste intensities. Sour, bitter, astringent, and bitter aftertaste intensities decreased as the sweet level increased, while sweetness increased. A less intense grapefruit flavor was perceived in the high sweet level beverages, compared to the low and medium sweet levels.

Very few two-way interactions were significant. The detailed tables for the significant interaction effects are presented in the Supplementary Material (Tables S2–S4). The bitter level x aroma level interaction effect (Table S2) was significant for the perception of the intensity of chemical aroma and overall flavor intensity, the bitter flavor, and the bitter aftertaste. A trend was observed in that the chemical aroma was more intensely perceived in the beverages with high aroma, although only significantly so in the low and high bitter samples and not in the medium bitter samples. The overall flavor intensity was significantly but slightly lower in the high aroma/medium bitter, compared to the low aroma/medium bitter sample. Aroma level did not affect the overall flavor perception at the low or high bitter levels. Bitter flavor and bitter aftertaste were notably less intense in the high aroma samples, compared to the low aroma samples, but only significantly so for the medium and high bitter level beverages.

The bitter level x color type interaction effect (Table S2) was significant for the bitter aftertaste intensity. However, bitter aftertaste was essentially driven more by the bitter level than the color type. The bitter level x sweet level interaction effect (Table S3) was significant only for the pungent aroma. A significantly lower pungent aroma was noted between the medium sweet and low sweet beverages, at the medium bitter level.

The aroma level x color interaction effect (Table S3) was significant only for bitter flavor intensity. At a low aroma level, no difference in bitter flavor intensity was found between the two colors. However, at the high aroma level, the yellow beverage was perceived as being significantly bitterer.

The sweet level x aroma level interaction effect (Table S4) was not significant for any of the sensory aroma attributes. A sweet level x color interaction effect (Table S4) was significant for the astringent and citrus flavor perception. While no significant differences were found between the red and yellow beverages at the medium sweet level, the red beverage was perceived as significantly more astringent at low sweet and high sweet levels. A similar effect was found for the citrus flavor, although the red beverages were found to have a more intense citrus flavor, only at the low sweet level.

**Figure 1:**
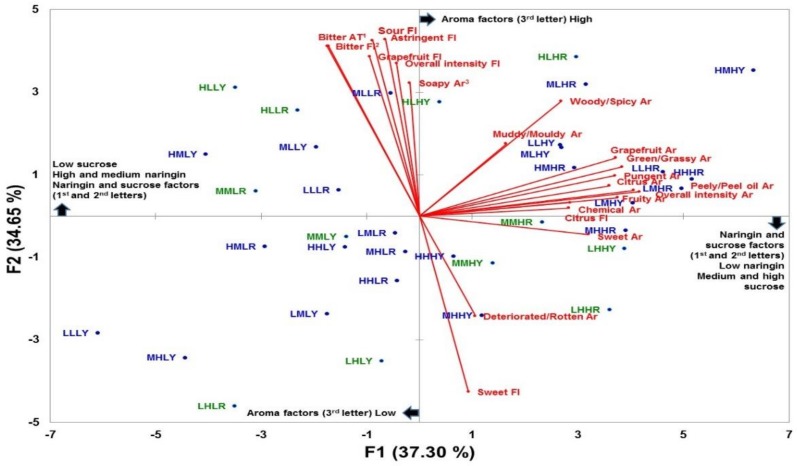
Principal Component Analysis (PCA) of the sensory profiles of the 36 grapefruit-like beverages. The vectors indicate the loadings for sensory attributes while the position of the sample codes indicate the score values. The four-letter codes indicate levels of naringin (1st letter: L = Low, M = Medium, or H = High), sucrose (2nd letter: L = Low, M = Medium, or H = High), aroma (3rd letter: L = Low, or H = High) and color (4th letter: R = red or Y = yellow). Sensory attributes ^1^AT = Aftertaste, ^2^Fl = Flavor, ^3^Ar = Aroma. Beverages in green font were selected for the consumer tests.

The multivariate differentiation of the beverages is presented in Figure 1 as a PCA map over a two-dimensional space. The first and second principal components (F1 and F2) explained 37% and 35%, respectively, of the variance across the samples. F1 clearly separated beverages based on intensity of overall aroma, peely/peel oil aroma, citrus aroma, sweet aroma, and pungent aroma. Beverages that were more intense in terms of the mentioned attributes are located on the right of the plot. Note that all of these beverages have an H as third letter, therefore, they have a high aroma level. The beverages with lower intensities are located on the left of the plot and notably has L as the third letter, therefore, with a low level aroma. F2 separated the beverages based on ‘taste’ perception, i.e., naringin (bitter)-sucrose (sweet) levels. Beverages with high and medium bitter levels and low sweet are positioned at the top, and beverages with low bitter level and medium and high sweet levels, are at the bottom. Beverages at the top, namely HLLY and HLLR with a high level of naringin and MLHY with a medium level, were characterized by more intense astringency, sour, and bitter tastes, and with grapefruit and overall flavor intensities. Beverages (e.g., LHHR) with a low naringin level (at the bottom), were characterized by a more intense sweet taste. The attributes citrus flavor, chemical aroma, and muddy/moldy aroma in the middle of the plot, did not discriminate beverages on the first two PCs.

### 3.2. Consumer Evaluation of the Grapefruit-Like Beverages

The effects of color, aroma level, and bitter/sweet levels of the grapefruit-like beverages on mean liking ratings for the color, aroma, and flavor, as evaluated by the consumers, are presented in Table 4. Two-way interaction effects were not significant. 

The standardized PLS regression coefficients for attributes as part of the prediction models are presented in Table 5. PLS regression (PLSR) models were used to predict liking of the color, aroma, and flavor of the 36 beverages, including the beverages that were not evaluated by consumers (Table 6). Expected errors of prediction for the models were low, lying between ±1.288 for the aroma model to ±2.458 for the color model, and ±2.678 for the flavor model, with a 95% confidence interval, indicating reliable prediction estimations of the liking variables.

Liking of the color of the red grapefruit-like beverages were rated, on average, slightly higher than the yellow ones (*p* < 0.05) (Table 4). Whether the beverage was colored yellow or red, it did not affect the liking of the aroma or the flavor. Predicted mean liking of the color for the highest and lowest liked of the 36 beverages differed, however, only by a maximum of 12.2 scale units (Table 6). Notably the research found no significant sensory attribute drivers for liking of the color of the grapefruit-like beverages (Table 5).

Liking of the aroma of beverages with a low added-aroma level, was higher (*p* < 0.05) than for those with a high added-aroma level (Table 4). Aroma level did not have an effect on the liking of the color of the beverage. Aroma level also did not affect the liking of the flavor of the beverage. The predicted mean liking of the aroma for the highest and the lowest liked beverages, differed by 16.5 scale units (Table 6). Main effects and squared effects are indicated as ‘^2^’. Positive attribute drivers for liking of the aroma of the grapefruit-like beverages were the square term of fruity aroma (noted fruity aroma^2^), citrus flavor, and sweet flavor, while negative drivers were sweet aroma ^2^, sweet flavor^2^, and pungent aroma (Table 5).

As expected, the level of the gustatory flavorants, the naringin, and the sucrose, did not affect the liking of the color of the beverages (Table 4). Surprisingly the non-volatile taste level did have a significant effect (*p* < 0.05) on the liking of the aroma of the beverages. The aroma of the most bitter/least sweet beverages was liked significantly less than the other two taste combination levels. Not surprisingly, liking of the flavor of the beverages decreased significantly (*p* < 001) as the bitter level increased and the sweet level decreased. Predicted mean ratings for liking of the flavor, the highest liked and the lowest liked of the 36 beverages, differed by 27.5 scale units. Positive drivers for liking of the flavor of the grapefruit-like beverages were sweet taste, squared term for chemical aroma (noted as ‘chemical aroma^2^), and citrus flavor intensities, while the negative drivers were intensity of soapy aroma, bitter aftertaste, and sour taste (Table 6).

## 4. Discussion

The research studied the effect of varying the bitterness, sweetness, color, and aroma intensity of grapefruit-like beverages on the cross-modal perception of sensory properties and its effects on consumer liking. A model grapefruit-like beverage standard formulation was created and a sensory lexicon with a total of 21 attributes and definitions were generated to characterize the aroma, the flavor, and the aftertaste of the grapefruit-like model beverage with variations in color, aroma, and gustatory flavorant levels.

Color hue of the grapefruit-like beverage affected the perception and description of the aroma and flavor sensory properties, as evaluated by the trained human panelists. Color of the beverages, and, in particular, the sample with the rose-red hue had a significant enhancing effect on perception of overall aroma intensity and grapefruit, deteriorated/rotten, muddy/moldy, fruity, and sweet aroma intensities. It also corresponded to the consumer liking—the red beverages were liked more than the yellow ones. The cross modal effect of the beverage color on aroma and flavor of the beverages, however, did not lead to significant differences in the liking of aroma or a liking of the flavor of the red and yellow beverages. The difference in methodology followed and the cognitive tasks employed by the two groups of panels might be the reason. When the group of consumers evaluated the liking of the color of the beverages, solely based on appearance, a slight but significant preference for the red-colored beverages was noted. This preference was solely driven by visual cues, since the consumers did not yet smell or taste the beverages. After smelling and tasting the beverages, it is likely that the opinion and preference might have changed, based on the cross-modal, color-aroma/flavor sensory interaction, as demonstrated by the results for the trained panel in this study. Considering that the consumers first evaluated the liking of the color, then the aroma (retronasally), and lastly the flavor (after consumption) of each sample, sequentially, it cannot be excluded that some form of learning, anticipation, and association might have occurred over the evaluation of the sequence of twelve samples, of which 50% were red and 50% were yellow.

A study [20] reported that the red color decreased the perception of the bitter taste sensitivity of a water solution. Coloring a clear bitter solution red, decreased the perception of the bitter taste, while the addition of yellow and green coloring had no such effect [9]. Other researchers [21] suggested that color-induced olfactory enhancement observed when odorous solutions are smelled orthonasally, might be the result of a conditioned olfactory percept caused by the color. Conditioned expectations predict that certain colors would be strongly associated with particular flavors, e.g., red with cherry, orange with orange, and green with lime [22]; yellow with lemon, blue with spearmint, and red with strawberry, raspberry, and cherry [23]. In South Africa, the location for the study, both yellow and red/pink grapefruit are marketed. The Star Ruby variety with a red color is the most planted (84%) grapefruit variety in South Africa, followed by the white variety Marsh (16%) (the juice of this type of grapes is pale yellow) [24]. In another study [25] it was found that the relationship of green and yellow colors in the lemon and lime-flavored sucrose solutions was altered; such color changes were found to have an impact on the perceived sweetness ratings. In another study, results showed that color–odor solution pairings were rated as having more intense odors with color cues than without, regardless of the color–odor pairing appropriateness [21]. This cross-modal effect presumably results from the color-cue setting up an expectation concerning the likely identity and intensity of a food or drink’s taste or flavor [20]. No significant sensory attribute drivers for liking of the color of the grapefruit-like beverages was identified, since the trained sensory panel did not evaluate the appearance attributes.

Aroma level added to the model beverage had a significant enhancing effect, on the majority of the aroma and flavor sensory attributes. The enhancement of overall aroma and characteristic aroma qualities, including citrus flavor, as a function of the level of aroma added, was expected and confirmed. When consumers evaluated liking of the aroma of the beverages, solely based on orthonasal inspection, surprisingly the beverages with low aroma were slightly preferred over those with high aroma. It is possible that the higher aroma level was more distinctive and clearly reminiscent of grapefruit and possibly evoked a stronger cue for those disliking grapefruit. An interesting and unexpected finding was the apparent suppression of bitter and astringent gustatory sensations, due to a higher load of olfactory stimuli (high aroma level). Previous studies have found that aroma–taste interactions can result in complicated changes in the perceived flavor. The addition of an aroma can, e.g., elevate the bitter-detection threshold [26,27]. The perceived intensity of tastes in solutions was increased by volatile compounds, especially when there was a logical association between them, such as between sweetness and fruitiness [28]. Apple and strawberry aromas evoked both sweetness and sourness. A study found that tasteless aromas, namely green tea and coffee, predominantly evoked bitterness, while the vanilla aroma predominantly evoked sweetness [29]. The grapefruit aroma consisted of a blend of caryophyllene, citral, nootkatone, and various aldehydes; octanal, nonanal, and decanal. No study could be found that specifically indicated that any of these compounds evoked bitterness. Nootkatone at the above threshold concentrations was reported as tasting bitter [30]. Consumption of a beverage results in the simultaneous perception of aroma and taste, coupled with tactile sensations, all of which contribute to an overall impression of flavor. Compounds that stimulate taste perception (e.g., naringin contributing a bitter taste) can increase the apparent intensity of aromas. In this study, the grapefruit flavor was enhanced by the naringin addition. The aroma compound (containing a citral component) of the grapefruit-like beverages had an enhancing effect on the citrus aroma intensity. An additive effect of the sweet components with citral or limonene volatiles having a ‘citrus’-like aroma was reported by [31] but was not observed in this study. The suppression of bitterness in the high aroma beverages, however, did not affect the liking of the flavor, since there was no difference found in the liking of the flavor of beverages with low or high aroma levels. Positive drivers for liking of the aroma of the grapefruit-like beverages were fruity aroma^2^, citrus flavor, and sweet flavor, while negative drivers were sweet aroma^2^, sweet flavor^2^, and pungent aroma.

The low bitter/high sweet beverages were preferred over the high bitter/low sweet samples. A study [32] reported that with an increase in the ratio of °Brix/acidity of reconstituted grapefruit juice, the consumer perception of sweetness increased and bitterness and aroma intensity decreased. Some bitterness in processed grapefruit products is acceptable for consumers, but excessive bitterness is one of the major consumer objections to such products [28,31]; this was confirmed in this study. The variation in sensitivity of the individual consumers to bitter compounds in grapefruit beverages could be explored further to identify whether subgroups might have different preferences. As expected, the contribution of varying concentrations of naringin affecting the bitterness of the grapefruit-like beverages did not have a significant effect on any of the aroma attributes. Similarly, [32] reported that consumers did not find any difference in aroma with increased levels of naringin in processed grapefruit juice. However, the concentration of bitterness of the grapefruit-like beverages had a significant effect on the flavor attributes (astringent, sweet, sour, bitter and grapefruit flavor, and the bitter aftertaste). A study [32] has also reported that an increase of limonin (also a bitter compound) in processed grapefruit juice, increased the perceived bitterness and tartness, while decreasing the sweetness.

In a previous study, an increase in the °Brix with sucrose, enhanced the taste of sweetness, and had a decreasing effect on the sour, bitter, astringent, and grapefruit flavors, and the bitter aftertaste. When sucrose was added to fruit juices, not only were the perceived levels of bitterness and sourness reduced (as was also found in this research) but the sweet aroma intensity rating also changed [16] (although this was not found here). Sucrose was also reported to mask the bitter taste of sinigrin, goitrin, and quinine [33]. In the complex beverage model, increasing sucrose did not have the often reported enhanced effect on the perceived fruity aroma. Increasing the sugar concentration of blueberry and cranberry fruit juices, increased their fruitiness (evaluated by sipping), even though no difference in aroma was perceived by sniffing alone [16]. Sucrose in the mouth significantly enhanced the “citrus” ratings, compared to when citral was inhaled alone [12]. Similarly, increases in the intensity of different ‘fruity’ aromas were perceived in a multichannel flavor delivery system [34], model dairy desserts [35], and custard desserts [36], when increasing the sweetness with sucrose. Sweet level also affected the soapy aroma of the grapefruit-like beverages. The reason for the effect on soapy aroma is unclear. It is possible that the aroma blend contributed a slight soapy aroma.

The effect of aroma level and color on the perceived sensory attributes, as observed in this study, are evidence of cross-modal sensory interactions. It was anticipated that the intensity and character of the aroma level of a grapefruit juice would increase the perception of the citrus flavor, a positive driver of grapefruit flavor liking and reduce the negative attributes, the bitter and astringent flavor, as well as the bitter aftertaste. Positive drivers for liking of the flavor of grapefruit-like beverages were the sweet taste, the chemical aroma, and the citrus flavor intensities, while negative drivers were intensity of soapy aroma, bitter aftertaste, and sour taste.

## 5. Conclusions

This study indicated that aroma, bitterness, and sweetness levels, and also product color (hue) influences the perception of grapefruit-like beverages, as well as their hedonic value. A grapefruit-like beverage model was created and a lexicon to describe the sensory properties of the cross-modal interaction of stimulus components of the model beverage was developed. From the descriptive sensory profiles, prediction models for liking of the color, aroma, and flavor of grapefruit-like beverages were developed. In the next phase, the models should be applied to a wide range of grapefruit juice samples to determine validity and reliability in real juices. The models can then be optimized for application in grapefruit quality control and product development programs.

## Figures and Tables

**Table 1:** Factorial design for the 36 grapefruit-like beverages.

Number	Code ^1^	Bitter Level Naringin mg/kg	Sweet Level Sucrose Brix	Aroma^2^ Level mg/kg	Color^3^
1	LMHR	158 low	10 medium	10 high.	Red
***2***	***MMHR***	***315 medium***	***10 medium***	***10 high***	***Red***
3	HMHR	473 high	10 medium	10 high.	Red
***4***	***LHHR***	***158 low***	***12 high***	***10 high***	***Red***
5	MHHR	315 medium	12 high	10 high.	Red
6	HHHR	473 high	12 high	10 high.	Red
7	LLHR	158 low	8 low	10 high.	Red
8	MLHR	315 medium	8 low	10 high.	Red
***9***	***HLHR***	***473 high***	***8 low***	***10 high***	***Red***
10	LMLR	158 low	10 medium	2.5 low	Red
***11***	***MMLR***	***315 medium***	***10 medium***	***2.5 low***	***Red***
12	HMLR	473 high	10 medium	2.5 low	Red
***13***	***LHLR***	***158 low***	***12 high***	***2.5 low***	***Red***
14	MHLR	315 medium	12 high	2.5 low	Red
15	HHLR	473 high	12 high	2.5 low	Red
16	LLLR	158 low	8 low	2.5 low	Red
17	MLLR	315 medium	8 low	2.5 low	Red
***18***	***HLLR***	***473 high***	***8 low***	***2.5 low***	***Red***
19	LMHY	158 low	10 medium	10 high.	Yellow
***20***	***MMHY***	***315 medium***	***10 medium***	***10 high.***	***Yellow***
21	HMHY	473 high	10 medium	10 high.	Yellow
*22*	***LHHY***	***158 low***	***12 high***	***10 high.***	***Yellow***
23	MHHY	315 medium	12 high	10 high.	Yellow
24	HHHY	473 high	12 high	10 high.	Yellow
25	LLHY	158 low	8 low	10 high.	Yellow
26	MLHY	315 medium	8 low	10 high.	Yellow
***27***	***HLHY***	***473 high***	***8 low***	***10 high.***	***Yellow***
28	LMLY	158 low	10 medium	2.5 low	Yellow
***29***	***MMLY***	***315 medium***	***10 medium***	***2.5 low***	***Yellow***
30	HMLY	473 high	10 medium	2.5 low	Yellow
***31***	***LHLY***	***158 low***	***12 high***	***2.5 low***	***Yellow***
32	MHLY	315 medium	12 high	2.5 low	Yellow
33	HHLY	473 high	12 high	2.5 low	Yellow
34	LLLY	158 low	8 low	2.5 low	Yellow
35	MLLY	315 medium	8 low	2.5 low	Yellow
***36***	***HLLY***	***473 high***	***8 low***	***2.5 low***	***Yellow***

^1^ Code: 1st letter = bitter level (High, Medium, or Low); 2nd letter = sweet level (High, Medium, or Low); 3rd letter = aroma level (High or Low); 4th letter = color (Red or Yellow). Samples in bold italics were used for consumer evaluation. ^2^ Aroma blend = Caryophyllene, citral, nootkatone, aldehyde C8 (octanal), aldehyde C9 (nonanal), aldehyde C10 (decanal). ^3^ Red color = 0.001% solution (30% Sunset yellow and 70% Ponceau red); Yellow color = 0.0125% Quinoline yellow.

**Table 2:** Definitions of attributes used for describing the aroma, flavor, and aftertaste of the grapefruit-like beverages.

Attribute	Definition (References Indicated Where Applicable)
Aroma	
Overall aroma intensity	The aroma of the beverage upon taking the first few sniffs
Citrus aroma	The aroma associated with the general impression of citrus fruits
Grapefruit aroma	The aroma of fresh grapefruit
Chemical aroma	A very general term associated with many different types of compounds, such as solvents and cleaning compounds
Deteriorated/rotten aroma	Aroma associated with rotten, deteriorated, and decayed fruit/material
Muddy/moldy aroma	Aromatic characteristic of damp soil, wet foliage, or slightly undercooked boiled potato
Fruity aroma	Aroma associated with a mixture of non-specific fruits (apples, pears, melons, and guava)
Green/grassy aroma	Aromatic characteristic of freshly cut leaves, grass, or green vegetables (green beans)
Peely/peel oil aroma	Aroma associated with grapefruit peel or skin; Ref: Grapefruit oil extracted from grapefruit
Soapy aroma	Aroma associated with unscented soap
Pungent aroma	Aroma causing a sharp sensation of the nasal mucous membranes; Ref: vinegar
Woody/spicy aroma	Aroma associated with dry, fresh-cut wood; balsamic or bark-like; Ref: 10 ppm alpha-humulone in water
Sweet aroma	Aroma associated with high sugar content vegetables;Ref: Freshly boiled sweet corn
Flavor	
Overall flavor	The intensity of the flavor that is released from the beverage upon taking the first sip
Sour taste	Basic taste on tongue stimulated by acids; Ref: citric acid in water
Sweet taste	Taste on the tongue stimulated by sugars;Ref: 5% sugar (sucrose) in water
Bitter taste	Taste on tongue stimulated by bitter solutions;Ref: 473 mg/kg naringin in water
Astringent flavor	The chemical feeling factor on the tongue or surface of the oral cavity described as puckering/dry and associated with tannins;Ref: Strong black tea
Citrus flavor	Flavor associated with the general impression of citrus fruits;Ref: Cut lemon fruit and lime cordial
Grapefruit flavor	The flavor of fresh grapefruit; Ref: Cut red and white grapefruit flesh
Bitter aftertaste	Bitter taste remaining in the mouth after swallowing the beverage

**Table 3:** Summary of the significance of varying the main effects of color hue, aroma, bitter, and sweet levels of a model grapefruit-like beverage, on the mean values ^1^ (±SEM) for sensory attributes as evaluated by a trained sensory panel (*n* = 16).

Attributes	Color ^2^			Aroma ^3^ mg/kg			Bitter (Naringin mg/kg)				Sweet (Sucrose mg/kg)			
	Red	Yellow		2.5	10		158 Low	315 Medium	473 High		8 Brix	10 Brix	12 Brix	
Overall aroma intensity	6.06a (0.05)	5.83b (0.05)	**	5.52b (0.05)	6.37a (0.05)	***	5.87a ² (0.06)	6.04a (0.06)	5.93a (0.06)	NS	5.96a (0.06)	5.91a (0.06)	5.96a (0.06)	NS
Citrus aroma	4.54a (0.05)	4.55a (0.05)	NS	4.23b (0.05)	4.86a (0.05)	***	4.51a (0.06)	4.58a (0.06)	4.55a (0.06)	NS	4.51a (0.06)	4.58a (0.06)	4.55a (0.06)	NS
Grapefruit aroma	4.40a (0.05)	4.15b (0.05)	**	4.06b (0.05)	4.49a (0.05)	***	4.28a (0.06)	4.23a (0.06)	4.32a (0.06)	NS	4.23a (0.06)	4.35a (0.06)	4.25a (0.06)	NS
Chemical aroma	4.09a (0.06)	4.03a (0.06)	NS	3.86b (0.06)	4.25a (0.06)	***	3.99a (0.07)	4.13a (0.07)	4.06a (0.07)	NS	4.06a (0.07)	4.03a (0.07)	4.09a (0.07)	NS
Deteriorated/rotten aroma	2.14a (0.04)	2.00b (0.04)	**	2.09a (0.04)	2.05a (0.04)	NS	2.08a (0.05)	2.04a (0.05)	2.09a (0.05)	NS	2.05a (0.05)	2.04a (0.05)	2.12a (0.05)	NS
Muddy/moldy aroma	2.20a (0.03)	2.09b (0.03)	**	2.09b (0.03)	2.20a (0.03)	**	2.12a (0.04)	2.13a (0.04)	2.18a (0.04)	NS	2.18a (0.04)	2.13a (0.04)	2.13a (0.04)	NS
Fruity aroma	3.93a (0.05)	3.78b (0.05)	*	3.71b (0.05)	4.00a (0.05)	***	3.89a (0.06)	3.83a (0.06)	3.85a (0.06)	NS	3.83a (0.06)	3.90a (0.06)	3.83a (0.06)	NS
Green/grassy aroma	3.13a (0.04)	3.14a (0.04)	NS	2.91b (0.04)	3.36a (0.04)	***	3.12a (0.05)	3.09a (0.05)	3.19a (0.05)	NS	3.12a (0.05)	3.18a (0.05)	3.10a (0.05)	NS
Peely/peel oil aroma	3.53a (0.05)	3.47a (0.05)	NS	3.20b (0.05)	3.79a (0.05)	***	3.53a (0.06)	3.47a (0.06)	3.49a (0.06)	NS	3.49a (0.06)	3.55a (0.06)	3.46a (0.06)	NS
Soapy aroma	3.29a (0.05)	3.23a (0.05)	NS	3.16b (0.05)	3.36a (0.05)	**	3.27a (0.06)	3.20a (0.06)	3.31a (0.06)	NS	3.41a (0.06)	4.33a (0.06)	3.04b (0.06)	**
Pungent aroma	3.16a (0.05)	3.11a (0.05)	NS	2.84b (0.05)	3.43a (0.05)	***	3.10a (0.06)	3.14a (0.06)	3.18a (0.06)	NS	3.17a (0.06)	3.13a (0.06)	3.11a (0.06)	NS
Woody/spicy aroma	2.49a (0.04)	2.40a (0.04)	NS	2.33b (0.04)	2.57a (0.04)	***	2.43a (0.04)	2.44a (0.04)	2.47a (0.04)	NS	2.48a (0.04)	2.48a (0.04)	2.37a (0.04)	NS
Sweet aroma	3.79a (0.05)	3.62b (0.05)	*	3.56b (0.05)	3.86a (0.05)	***	3.73a (0.06)	3.69a (0.06)	3.70a (0.06)	NS	3.69a (0.06)	3.72a (0.06)	3.72a (0.06)	NS
Overall flavor intensity	6.39a (0.05)	6.34a (0.05)	NS	6.37a (0.05)	6.36a (0.05)	NS	6.14b (0.06)	6.51a (0.06)	6.46a (0.06)	***	6.41a (0.06)	6.29a (0.06)	6.40a (0.06)	NS
Sour flavor	5.09a (0.06)	5.17a (0.06)	NS	5.14a (0.06)	5.11a (0.06)	NS	4.90b (0.08)	5.08b (0.08)	5.40a (0.08)	***	5.93a (0.08)	5.07b (0.08)	4.38c (0.08)	***
Sweet flavor	4.48a (0.05)	4.43a (0.05)	NS	4.38a (0.05)	4.53a (0.05)	NS	4.64a (0.06)	4.51a (0.06)	4.21b (0.06)	***	3.04c (0.06)	4.50b (0.06)	5.82a (0.06)	***
Bitter flavor	4.56a (0.07)	4.48a (0.07)	NS	4.77a (0.07)	4.27b (0.07)	***	3.94c (0.08)	4.46b (0.08)	5.17a (0.08)	***	5.24a (0.08)	4.43b (0.08)	3.89c (0.08)	***
Astringent flavor	4.95a (0.06)	4.81a (0.06)	NS	4.97a (0.06)	4.79b (0.06)	*	4.62b (0.07)	4.91a (0.07)	5.12a (0.07)	***	5.35a (0.07)	4.88b (0.07)	4.41c (0.07)	***
Citrus flavor	4.47a (0.05)	4.43a (0.05)	NS	4.30b (0.05)	4.60a (0.05)	***	4.52a (0.06)	4.44a (0.06)	4.39a (0.06)	NS	4.43a (0.06)	4.45a (0.06)	4.48a (0.06)	NS
Grapefruit flavor	4.53a (0.05)	4.36b (0.05)	*	4.49a (0.05)	4.40a (0.05)	NS	4.19b (0.07)	4.41b (0.07)	4.73a (0.07)	***	4.53a (0.07)	4.54a (0.07)	4.26b (0.07)	**
Bitter aftertaste	4.32a (0.07)	4.26a (0.07)	NS	4.49a (0.07)	4.08b (0.07)	***	3.69c (0.08)	4.26b (0.08)	4.91a (0.08)	***	4.90a (0.08)	4.25b (0.08)	3.71c (0.08)	***

^1^ Attribute intensity scale from ‘not intense’ (0) to ‘very intense’ (10); ^2^ Red = 0.001% solution (30% Sunset yellow and 70% Ponceau red); Yellow = 0.0125% Quinoline yellow. ^3^ Aroma blend {caryophyllene, citral, nootkatone, aldehyde C8 (octanal), aldehyde C9 (nonanal), and aldehyde C10 (decanal)}. abc—different letters indicate significantly different mean values across design variable levels. Means represent the average of duplicate ratings by 16 panelists; * *p* ≤ 0.05, ** *p* ≤ 0.01, *** *p* ≤ 0.0001; NS = not significantly different.

**Table 4:** The effect of varying the color, aroma, and bitter/sweet gustatory flavorants on mean liking ratings ^1^ (±standard deviation) for color, aroma, and flavor of grapefruit-like beverages by (*n* = 90) consumers.

	Color ^2^			Aroma ^3^ mg/kg ^3^			Bitter-Sweet Naringin mg/kg /Sucrose Brix			
	Red	Yellow		2.5	10 mg/kg		158/12 Low/High	315/10 Medium/Medium	473/8 High/Low	
Liking of color	64b (30)	60a (30)	**	63a (30)	61a (31)	NS	62a ^1^ (30)	62a (31)	61a (30)	NS
Liking of aroma	51a (30)	51a (30)	NS	53a (29)	49b (31)	***	50ab (30)	53a (30)	49b (29)	*
Liking of flavor	45a (33)	45a (34)	NS	45a (34)	45a (34)	NS	55a (33)	46b (33)	34c (32)	***

^1^ Simplified Labelled Affective Magnitude Scale (SLAM); 0 = greatest imaginable dislike, 100 = greatest imaginable liking. abc—different letters indicate significantly different mean values across the design variable levels. NS = not significant, * *p* ≤ 0.05, ** *p* ≤ 0.01, *** *p* ≤ 0.001. ^2^ Red = 0.001% solution (30% Sunset yellow and 70% Ponceau red); Yellow = 0.0125% Quinoline yellow.^. 3^ Aroma blend {caryophyllene, citral, nootkatone, aldehyde C8 (octanal), aldehyde C9 (nonanal), and aldehyde C10 (decanal)}.

**Table 5:** Standardized partial least squares (PLS) regression coefficients for factors to summarize the relationship between predictors (X, consumer liking variables) and Y, sensory response variables. Only selected important variables (main effects and squared effects, noted as ‘^2^’) from the refined models are shown.

Liking of the Color R^2^ = 0.871		Liking of the Aroma R^2^ = 0.970		Liking of the Flavor R^2^ = 0.982	
Overall aroma intensity ^2^	0.23	Fruity aroma ^2^	0.08	Sweet aroma	0.16
Citrus aroma ^2^	0.16	Citrus flavor	0.03	Chemical aroma ^2^	0.15
Sweet aroma	0.16	Sweet flavor	0.03	Citrus flavor	0.12
Astringent flavor ^2^	0.15	Astringent flavor	0.02	Deteriorated/rotten aroma	0.03
Green/grassy aroma ^2^	0.10	Grapefruit flavor	0.02	Green/grassy aroma ^2^	−0.01
Fruity aroma	0.00	Bitter aftertaste	−0.01	Greed/grassy aroma	−0.01
Overall aroma intensity	0.00	Bitter flavor	−0.01	Chemical aroma	−0.01
Grapefruit aroma	0.00	Sour flavor	−0.02	Woody/spicy aroma ^2^	−0.04
Green/grassy aroma	−0.01	Overall flavor intensity	−0.04	Overall flavor intensity	−0.04
Astringent flavor	−0.04	Deteriorated/rotten aroma	−0.05	Bitter flavor	−0.07
Peely/peel oil aroma	−0.04	Soapy aroma	−0.05	Grapefruit flavor	−0.07
Pungent aroma	−0.05	Chemical aroma	−0.06	Woody/spicy aroma	−0.08
Citrus aroma	−0.06	Woody/spicy aroma	−0.06	Fruity aroma	−0.08
Citrus flavor	−0.07	Sweet aroma	−0.07	Muddy/moldy aroma	−0.10
Muddy/moldy aroma	−0.08	Fruity aroma	−0.07	Bitter flavor	−0.11
Woody/spicy aroma	−0.09	Peely/peel oil aroma	−0.08	Astringent flavor	−0.11
Soapy aroma	−0.13	Pungent aroma ^2^	−0.08	Sour flavor	−0.12
Chemical aroma ^2^	−0.18	Citrus aroma	−0.09	Bitter aftertaste	−0.12
Chemical aroma	−0.19	Grapefruit aroma	−0.09	Soapy aroma	−0.19
		Overall aroma intensity	−0.10		
		Green/grassy aroma	−0.11		
		Muddy/moldy aroma	−0.12		
		Bitter flavor	−0.14		
		Pungent aroma	−0.14		
		Sweet flavor ^2^	−0.16		
		Sweet aroma ^2^	−0.17		

**Table 6:** Partial least square regression (PLSR) model predicted liking ratings for color, aroma, and flavor of the grapefruit-like beverages.

		Color Liking		Aroma Liking		Flavor Liking	
Number ^1^	Code ^2^	Observed ^3^	Predicted	Observed	Predicted	Observed	Predicted
***2***	***MMHR***	***61ab (31)***	***61***	***51ab (30)***	***51***	***44abc (31)***	***45***
***4***	***LHHR***	***64ab (29)***	***64***	***45b (30)***	***45***	***54a (32)***	***52***
***9***	***HLHR***	***63ab (33)***	***64***	***47ab (30)***	***47***	***35bc (33)***	***36***
***11***	***MMLR***	***66a (29)***	***64***	***57a (29)***	***56***	***48a (33)***	***48***
***13***	***LHLR***	***67a (30)***	***68***	***55ab (29)***	***55***	***55a (34)***	***55***
***18***	***HLLR***	***62ab (30)***	***61***	***51ab (29)***	***50***	***32c (29)***	***32***
***20***	***MMHY***	***62ab (32)***	***61***	***52ab (29)***	***52***	***48a (35)***	***48***
***22***	***LHHY***	***55b (32)***	***56***	***48ab (33)***	***48***	***54a (34)***	***54***
***27***	***HLHY***	***59ab (30)***	***60***	***49ab (31)***	***49***	***35bc (34)***	***34***
***29***	***MMLY***	***60ab (32)***	***60***	***54ab (31)***	***54***	***46ab (34)***	***47***
***31***	***LHLY***	***61ab (29)***	***62***	***52ab (29)***	***53***	***55a (33)***	***56***
***36***	***HLLY***	***61ab (28)***	***62***	***50ab (28)***	***51***	***35bc (32)***	***34***
1	LMHR		59		44		41
3	HMHR		62		49		41
5	MHHR		63		47		47
6	HHHR		60		46		45
7	LLHR		62		46		41
8	MLHR		60		47		38
10	LMLR		61		53		47
12	HMLR		64		54		45
14	MHLR		61		52		50
15	HHLR		63		52		48
16	LLLR		62		53		43
17	MLLR		59		48		32
19	LMHY		59		49		44
21	HMHY		62		41		38
23	MHHY		62		52		57
24	HHHY		62		52		51
25	LLHY		60		48		36
26	MLHY		61		45		40
28	LMLY		63		55		56
30	HMLY		62		54		40
32	MHLY		66		55		56
33	HHLY		63		52		46
34	LLLY		68		57		53
35	MLLY		60		52		39

^1^ Refer to Table 1 for number. ^2^ Code: 1st letter = bitter level (High, Medium, or Low); 2nd letter = sweet level (High, Medium, or Low); 3rd letter = aroma level (High or Low); 4th letter = color (Red or Yellow). Samples in bold italic were used for consumer evaluation. ^3^ Values are means (± standard deviation); Observed means in a column with different letters are significantly different (*p* < 0.05).

## Supplementary Materials

The following are available online at http://www.mdpi.com/2072-6643/11/2/464/s1. Table S1: Physico-chemical characterization (means ± standard deviation) of the 36 grapefruit-like beverages. Table S2: Summary of sensory attribute mean values1 [± standard error of means (SEM)] and significance of bitter x aroma and bitter x color two-way ANOVA interactions of the model grapefruit-like beverages as evaluated by a trained sensory panel (*n* = 16). Table S3: Summary of sensory attribute mean values1 [± standard error of means (SEM)] and significance of bitter x sweet and aroma x color two-way ANOVA interactions of the model grapefruit-like beverages as evaluated by a trained sensory panel (*n* = 16). Table S4: Summary of sensory attribute mean values1 [± standard error of means (SEM)] and significance of sweet x aroma and sweet x color two-way ANOVA interactions of the model grapefruit-like beverages as evaluated by a trained sensory panel (*n* = 16).
