# Supplementary material for: Effects of Varying the Color, Aroma, Bitter, and Sweet Levels of a Grapefruit-Like Model Beverage on the Sensory Properties and Liking of the Consumer
Source: Nutrients. 2019 Feb 22;11(2):464. doi: 10.3390/nu11020464 (PMC6413041; doi:10.3390/nu11020464)
Supplement: Supplementary file 1 [file nutrients-11-00464-s001.pdf]

**Table S1**. Physico-chemical characterization (means ±standard deviation) of the 36 grapefruit-like beverages.

| No | Sample code [2] | Brix | TA % | Brix/TA Ratio | Sucrose g/100 g | Fructose g/100 g | Glucose g/100 g | L | a | b |
|---|---|---|---|---|---|---|---|---|---|---|
| 1 | LMHR | 10.80b (0.01) | 1.26cd (0.01) | 8.75b (0.05) | 1.54cd (0.03) | 4.20b (0.11) | 2.75a (0.13) | 25.20ijkl (0.02) | 2.28bc (0.01) | -0.26 l (0.03) |
| 2 | MMHR | 10.79b (0.02) | 1.26cd (0.01) | 8.77b (0.07) | 1.52cd (0.00) | 4.44ab (0.06) | 2.82a (0.01) | 24.87lmn (0.05) | 2.17def (0.06) | -0.23ijkl (0.02) |
| 3 | HMHR | 10.81b (0.02) | 1.27bc (0.01) | 8.74b (0.04) | 1.46d (0.02) | 4.43ab (0.08) | 2.87a (0.01) | 24.63n (0.07) | 1.84jk (0.01) | -0.43o (0.01) |
| 4 | LHHR | 12.74a (0.01) | 1.23e (0.01) | 10.58a (0.05) | 3.52b (0.07) | 4.31ab (0.01) | 2.72a (0.04) | 25.16ijklm (0.03) | 2.43a (0.06) | -0.09fg (0.00) |
| 5 | MHHR | 12.80a (0.04) | 1.23e (0.07) | 10.91a (0.64) | 3.67ab (0.25) | 4.36ab (0.28) | 2.83a (0.13) | 25.13jklm (0.03) | 2.19cde (0.04) | -0.26l (0.02) |
| 6 | HHHR | 12.80a (0.03) | 1.23e (0.01) | 10.57a (0.06) | 3.62ab (0.03) | 4.33ab (0.14) | 2.79a (0.02) | 24.81mn (0.20) | 2.02gh (0.04) | -0.36mno (0.03) |
| 7 | LLHR | 8.83c (0.01) | 1.29a (0.01) | 7.03c (0.04) | 0.00e (0.00) | 4.46ab (0.15) | 2.84a (0.01) | 24.95klmn (0.95) | 2.59a (0.27) | -0.17hij (0.04) |
| 8 | MLHR | 8.83c (0.00) | 1.29a (0.01) | 7.03c (0.03) | 0.00e (0.00) | 4.42ab (0.12) | 2.79a (0.09) | 25.15ijklm (0.31) | 2.17def (0.03) | -0.24jkl (0.03) |
| 9 | HLHR | 8.78c (0.05) | 1.29a (0.01) | 7.01c (0.09) | 0.00e (0.00) | 4.45ab (0.11) | 2.82a (0.01) | 25.35ghij (0.06) | 1.97hi (0.03) | -0.38no (0.02) |
| 10 | LMLR | 10.78b (0.06) | 1.26cd (0.00) | 8.76b (0.05) | 1.63cd (0.06) | 4.35ab (0.07) | 2.78a (0.03) | 25.15ijklm (0.30) | 2.25bcd (0.07) | -0.14gh (0.07) |
| 11 | MMLR | 10.76b (0.04) | 1.25cd (0.08) | 8.78b (0.81) | 1.63cd (0.01) | 4.41ab (0.04) | 2.79a (0.04) | 25.34ghij (0.02) | 2.16def (0.02) | -0.25kl (0.01) |
| 12 | HMLR | 10.77b (0.03) | 1.26cd (0.00) | 8.75b (0.03) | 1.63cd (0.03) | 4.35ab (0.07) | 2.80a (0.12) | 25.15ijklm (0.01) | 1.81k (0.04) | -0.43no (0.02) |
| 13 | LHLR | 12.73a (0.01) | 1.23e (0.00) | 10.54a (0.01) | 3.71ab (0.01) | 4.31ab (0.01) | 2.79a (0.06) | 25.51ghi (0.02) | 2.31b (0.01) | -0.15ghi (0.01) |
| 14 | MHLR | 12.92a (0.24) | 1.23e (0.00) | 10.54a (0.20) | 3.74ab (0.49) | 4.35ab (0.50) | 2.81a (0.33) | 25.42ghij (0.01) | 2.12efg (0.05) | -0.27l (0.02) |
| 15 | HHLR | 12.71a (0.03) | 1.23e (0.00) | 10.53a (0.02) | 3.71ab (0.01) | 4.27ab (0.02) | 2.80a (0.03) | 25.32ghij (0.05) | 1.91ijk (0.02) | -0.39no (0.01) |
| 16 | LLLR | 8.79c (0.03) | 1.29a (0.01) | 7.03c (0.04) | 0.00e (0.00) | 4.50ab (0.17) | 2.83a (0.06) | 25.57gh (0.04) | 2.08fg (0.04) | -0.28lm (0.02) |
| 17 | MLLR | 8.83c (0.02) | 1.29a (0.01) | 7.06c (0.04) | 0.00e (0.00) | 4.44ab (0.12) | 2.81a (0.07) | 25.23hijkl (0.07) | 1.92ij (0.04) | -0.37no (0.02) |
| 18 | HLLR | 8.78c (0.01) | 1.29a (0.01) | 7.03c (0.04) | 0.00e (0.00) | 4.35ab (0.02) | 2.76a (0.04) | 25.29hijk (0.02) | 1.95hi (0.03) | -0.35mn (0.03) |
| 19 | LMHY | 10.79b (0.00) | 1.26cd (0.00) | 8.77b (0.00) | 1.63cd (0.05) | 4.40ab (0.21) | 2.76a (0.08) | 26.60abc (0.06) | -0.20pqrs (0.03) | 0.41ab (0.02) |
| 20 | MMHY | 10.77b (0.01) | 1.26cd (0.00) | 8.75b (0.01) | 1.64cd (0.31) | 4.28ab (0.60) | 2.76a (0.36) | 26.59abc (0.01) | -0.23rs (0.02) | 0.39abc (0.01) |
| 21 | HMHY | 10.72b (0.05) | 1.25cd (0.01) | 8.76b (0.07) | 1.64cd (0.00) | 4.43ab (0.03) | 2.81a (0.05) | 26.19de (0.06) | -0.08m (0.01) | 0.16e (0.01) |
| 22 | LHHY | 12.77a (0.02) | 1.23e (0.00) | 10.58a (0.02) | 3.67ab (0.11) | 4.37ab (0.05) | 2.75a (0.05) | 26.63abc (0.06) | -0.25rs (0.01) | 0.46a (0.02) |
| 23 | MHHY | 12.76a (0.03) | 1.23e (0.00) | 10.57a (0.03) | 3.79a (0.09) | 4.36ab (0.07) | 2.78a (0.01) | 26.26cde (0.06) | -0.10mno (0.02) | 0.20e (0.02) |
| 24 | HHHY | 12.75a (0.06) | 1.23e (0.00) | 10.56a (0.05) | 3.70ab (0.06) | 4.30ab (0.07) | 2.77a (0.02) | 26.29bcde (0.06) | -0.10mnop (0.01) | 0.20e (0.02) |
| 25 | LLHY | 8.81c (0.02) | 1.29a (0.01) | 7.05c (0.04) | 0.00e (0.00) | 4.44ab (0.10) | 2.82a (0.02) | 26.59abc (0.04) | -0.22rs (0.04) | 0.38abc (0.03) |
| 26 | MLHY | 8.82c (0.01) | 1.29a (0.00) | 7.04c (0.01) | 0.00e (0.00) | 4.33ab (0.15) | 2.75a (0.06) | 26.38abcd (0.05) | -0.16mnopqr (0.02) | 0.29d (0.02) |
| 27 | HLHY | 8.73c (0.10) | 1.28ab (0.00) | 7.02c (0.08) | 0.00e (0.00) | 4.52ab (0.03) | 2.84a (0.04) | 25.99ef (0.02) | 0.02l (0.04) | -0.04f (0.01) |
| 28 | LMLY | 10.81b (0.01) | 1.26cd (0.00) | 8.79b (0.00) | 1.71c (0.08) | 4.54a (0.04) | 2.86a (0.05) | 26.69a (0.08) | -0.23rs (0.03) | 0.43a (0.04) |
| 29 | MMLY | 10.78b (0.02) | 1.25d (0.00) | 8.83b (0.01) | 1.62cd (0.06) | 4.49ab (0.02) | 2.88a (0.08) | 26.42abcd (0.08) | -0.19nopqrs (0.02) | 0.30d (0.00) |
| 30 | HMLY | 10.77b (0.01) | 1.25d (0.00) | 8.82b (0.01) | 1.60cd (0.02) | 4.39ab (0.07) | 2.81a (0.02) | 25.67fg (0.01) | 0.09l (0.02) | -0.17hijk (0.01) |
| 31 | LHLY | 12.75a (0.03) | 1.23e (0.01) | 10.60a (0.03) | 3.61ab (0.07) | 4.30ab (0.09) | 2.76a (0.02) | 26.62abc (0.01) | -0.26s (0.03) | 0.43a (0.01) |
| 32 | MHLY | 12.72a (0.08) | 1.23e (0.01) | 10.57a (0.10) | 3.73ab (0.17) | 4.35ab (0.14) | 2.83a (0.08) | 26.45abcd (0.10) | -0.15mnopqr (0.02) | 0.33bcd (0.04) |
| 33 | HHLY | 12.81a (0.02) | 1.23e (0.01) | 10.58a (0.04) | 3.67ab (0.01) | 4.41ab (0.03) | 2.79a (0.02) | 26.29bcde (0.04) | -0.09mn (0.02) | 0.20e (0.03) |
| 34 | LLLY | 8.80c (0.01) | 1.29a (0.00) | 7.02c (0.01) | 0.00e (0.00) | 4.37ab (0.07) | 2.77a (0.03) | 26.63ab (0.04) | -0.21qrs (0.01) | 0.38abc (0.01) |
| 35 | MLLY | 8.80c (0.03) | 1.28a (0.01) | 7.06c (0.05) | 0.00e (0.00) | 4.26ab (0.02) | 2.75a (0.00) | 26.29bcde (0.05) | -0.12mnopq (0.02) | 0.19e (0.01) |
| 36 | HLLY | 8.71c (0.01) | 1.28ab (0.00) | 7.01c (0.01) | 0.00e (0.00) | 4.29ab (0.12) | 2.70a (0.01) | 26.52abcd (0.07) | -0.19opqrs (0.02) | 0.32cd (0.03) |

[2] Refer to Table 1, Mean values in a column with different letters are significantly different ($p < 0.05$).

**Table S2.** Summary of sensory attribute mean values [1] [± standard error of means (SEM)] and significance of bitter x aroma and bitter x color two-way ANOVA interactions of the model grapefruit-like beverages as evaluated by a trained sensory panel ($n$ = 16).

| Attributes | Bitter (Naringin mg/kg) x Aroma (mg/kg) [2] | | | | | | | Bitter (Naringin mg/kg) x Color[3] | | | | | | |
|---|---|---|---|---|---|---|---|---|---|---|---|---|---|---|
| | Bitter (Naringin mg/kg) | | | | | | | Bitter (Naringin mg/kg) | | | | | | |
| | 158 (low) | | 315 (medium) | | 473 (high) | | | 158 (low) | | 315 (medium) | | 473 (high) | | |
| | Aroma(mg/kg) | | | | | | | Color | | | | | | |
| | 2.5 Low | 10 High | 2.5 Low | 10 High | 2.5 Low | 10 High | | Red | Yellow | Red | Yellow | Red | Yellow | |
| **Overall aroma intensity** | 5.40a (0.08) | 6.33a (0.08) | 5.71a (0.08) | 6.37a (0.08) | 5.46a (0.08) | 6.39a (0.08) | NS | 5.94a (0.08) | 5.79a (0.08) | 6.18a (0.08) | 5.89a (0.08) | 6.05a (0.08) | 5.81a (0.08) | NS |
| **Citrus aroma** | 4.19a (0.08) | 4.83a (0.08) | 4.33a (0.08) | 4.82a (0.08) | 4.17a (0.08) | 4.93a (0.08) | NS | 4.45a (0.08) | 4.57a (0.08) | 4.67a (0.08) | 4.49a (0.08) | 4.50a (0.08) | 4.60a (0.08) | NS |
| **Grapefruit aroma** | 4.01a (0.08) | 4.54a (0.08) | 4.07a (0.08) | 4.39a (0.08) | 4.10a (0.08) | 4.53a (0.08) | NS | 4.43a (0.08) | 4.13a (0.08) | 4.36a (0.08) | 4.10a (0.08) | 4.40a (0.08) | 4.23a (0.08) | NS |
| **Chemical aroma** | **3.65c (0.10)** | **4.34a (0.10)** | **4.04a (0.10)** | **4.22a (0.10)** | **3.91b (0.10)** | **4.20a (0.10)** | * | 4.04a (0.10) | 3.94a (0.10) | 4.18a (0.10) | 4.08a (0.10) | 4.04a (0.10) | 4.08a (0.10) | NS |
| **Deteriorated/rotten aroma** | 2.07a (0.06) | 2.09a (0.06) | 2.08a (0.06) | 1.99a (0.06) | 2.12a (0.06) | 2.06a (0.06) | NS | 2.18a (0.06) | 1.98a (0.06) | 2.05a (0.06) | 2.03a (0.06) | 2.19a (0.06) | 1.99a (0.06) | NS |
| **Muddy/mouldy aroma** | 2.02a (0.05) | 2.23a (0.05) | 2.12a (0.05) | 2.15a (0.05) | 2.13a (0.05) | 2.23a (0.05) | NS | 2.19a (0.05) | 2.06a (0.05) | 2.20a (0.05) | 2.07a (0.05) | 2.22a (0.05) | 2.13a (0.05) | NS |
| **Fruity aroma** | 3.70a (0.08) | 4.08a (0.08) | 3.69a (0.08) | 3.98a (0.08) | 3.74a (0.08) | 3.95a (0.08) | NS | 3.93a (0.08) | 3.85a (0.08) | 3.89a (0.08) | 3.78a (0.08) | 3.97a (0.08) | 3.72a (0.08) | NS |
| **Green/grassy aroma** | 2.85a (0.07) | 3.40a (0.07) | 2.92a (0.07) | 3.26a (0.07) | 2.96a (0.07) | 3.43a (0.07) | NS | 3.15a (0.07) | 3.09a (0.07) | 3.10a (0.07) | 3.08a (0.07) | 3.15a (0.07) | 3.24a (0.07) | NS |
| **Peely/peel oil aroma** | 3.17a (0.08) | 3.90a (0.08) | 3.22a (0.08) | 3.72a (0.08) | 3.22a (0.08) | 3.77a (0.08) | NS | 3.56a (0.08) | 3.50a (0.08) | 3.49a (0.08) | 3.45a (0.08) | 3.54a (0.08) | 3.45a (0.08) | NS |
| **Soapy aroma** | 3.08a (0.09) | 3.45a (0.09) | 3.12a (0.09) | 3.28a (0.09) | 3.28a (0.09) | 3.34a (0.09) | NS | 3.25a (0.09) | 3.28a (0.09) | 3.30a (0.09) | 3.10a (0.09) | 3.31a (0.09) | 3.32a (0.09) | NS |
| **Pungent aroma** | 2.73a 0.08) | 3.47a (0.08) | 2.95a (0.08) | 3.32a (0.08) | 2.85a (0.08) | 3.50a (0.08) | NS | 3.12a (0.08) | 3.08a (0.08) | 3.19a (0.08) | 3.09a (0.08) | 3.18a (0.08) | 3.18a (0.08) | NS |
| **Woody/spicy aroma** | 2.27a (0.06) | 2.59a (0.06) | 2.35a (0.06) | 2.53a (0.06) | 2.36a (0.06) | 2.58 (0.06) | NS | 2.43a (0.06) | 2.43a (0.06) | 2.53a (0.06) | 2.35a (0.06) | 2.52a (0.06) | 2.42a (0.06) | NS |
| **Sweet aroma** | 3.53a (0.09) | 3.93a (0.09) | 3.56a (0.09) | 3.82a (0.09) | 3.58a (0.09) | 3.83 (0.09) | NS | 3.85a (0.09) | 3.61a (0.09) | 3.72a (0.09) | 3.66a (0.09) | 3.81a (0.09) | 3.60a (0.09) | NS |
| **Overall flavour intensity** | **6.06c (0.08)** | **6.22bc (0.08)** | **6.62a (0.08)** | **6.39b (0.08)** | **6.43a (0.08)** | **6.48a (0.08)** | * | 6.19a (0.08) | 6.09a (0.08) | 6.62a (0.08) | 6.40a (0.08) | 6.37a (0.08) | 6.54a (0.08) | NS |
| **Sour flavour** | 4.83a (0.11) | 4.98a (0.11) | 5.20a (0.11) | 4.95a (0.11) | 5.40a (0.11) | 5.41a (0.11) | NS | 4.86a (0.11) | 4.94a (0.11) | 5.08a (0.11) | 5.07a (0.11) | 5.32a (0.11) | 5.48a (0.11) | NS |

| | | | | | | | | | | | | | | |
|---|---|---|---|---|---|---|---|---|---|---|---|---|---|---|
| **Sweet flavour** | 4.53a (0.09) | 4.75a (0.09) | 4.45a (0.09) | 4.58a (0.09) | 4.18a (0.09) | 4.24a (0.09) | NS | 4.65a (0.09) | 4.63a (0.09) | 4.59a (0.09) | 4.43a (0.09) | 4.19a (0.09) | 4.23a (0.09) | NS |
| **Bitter flavour** | **4.00d (0.12)** | **3.88d (0.12)** | **4.87bc (0.12)** | **4.05d (0.12)** | **5.44a (0.12)** | **4.89b (0.12)** | * | 4.01a (0.12) | 3.86a (0.12) | 4.58a (0.12) | 4.34a (0.12) | 5.08a (0.12) | 5.25a (0.12) | NS |
| **Astringent flavour** | 4.60a (0.10) | 4.63a (0.10) | 5.04a (0.10) | 4.76a (0.10) | 5.25a (0.10) | 4.98a (0.10) | NS | 4.71a (0.10) | 4.52a (0.10) | 5.01a (0.10) | 4.80a (0.10) | 5.12a (0.10) | 5.11a (0.10) | NS |
| **Citrus flavour** | 4.42a (0.08) | 4.62a (0.08) | 4.29a (0.08) | 4.59a (0.08) | 4.20a (0.08) | 4.57a (0.08) | NS | 4.54a (0.08) | 4.50a (0.08) | 4.41a (0.08) | 4.48a (0.08) | 4.45a (0.08) | 4.32a (0.08) | NS |
| **Grapefruit flavour** | 4.17a (0.09) | 4.21a (0.09) | 4.57a (0.09) | 4.25a (0.09) | 4.73a (0.09) | 4.73a (0.09) | NS | 4.37a (0.09) | 4.01a (0.09) | 4.49a (0.09) | 4.33a (0.09) | 4.73a (0.09) | 4.72a (0.09) | NS |
| **Bitter aftertaste** | **3.70c (0.12)** | **3.68c (0.12)** | **4.54b (0.12)** | **3.97c (0.12)** | **5.23a (0.12)** | **4.60b (0.12)** | * | **3.79bc (0.12)** | **3.59c (0.12)** | **4.40b (0.12)** | **4.11b (0.12)** | **4.77a (0.12)** | **5.06a (0.12)** | * |

[1] Mean values in a row for a specific two-way interaction effect printed in bold and with different letters are significantly different;, * $p \leq 0.05$, ** $p \leq 0.01$, *** $p \leq 0.0001$; NS = not significantly different Attribute intensity scale (0–10 cm) ranging from 'not intense' (0) to 'very intense' (10); [2]Aroma blend [Caryophylene, citral, nootkatone, aldehyde C8 (octanal), aldehyde C9 (nonanal), aldehyde C10 (decanal)] [3]Red color = 0.001% solution (30 % Sunset yellow and 70 % Ponceau red); Yellow color = 0.0125 % Quinoline yellow.

**Table S3.** Summary of sensory attribute mean values [1] [± standard error of means (SEM)] and significance of bitter x sweet and aroma x color two-way ANOVA interactions of the model grapefruit-like beverages as evaluated by a trained sensory panel ($n$ = 16).

| **Attributes** | **Bitter (Naringin mg/kg) x Sweet (°Brix)** | | | | | | | | | | **Aroma mg/kg[2]x Color[3]** | | | | |
|---|---|---|---|---|---|---|---|---|---|---|---|---|---|---|---|
| | **Bitter (Naringin mg/kg)** | | | | | | | | | | **Aroma mg/kg** | | | | |
| | **158 (low)** | | | **315 (medium)** | | | **473 (high)** | | | | **2.5 (low)** | | **10 (high)** | | |
| | **Sweet (Brix)** | | | | | | | | | | **Color** | | | | |
| | **8 Low** | **10 Medium** | **12 High** | **8 Low** | **10 Medium** | **12 High** | **8 Low** | **10 Medium** | **12 High** | | **Red** | **Yellow** | **Red** | **Yellow** | |
| **Overall aroma intensity** | 5.90a[2] (0.10) | 5.90a (0.10) | 5.80a (0.10) | 6.20a (0.10) | 5.89a (0.10) | 6.03a (0.10) | 5.80a (0.10) | 5.94a (0.10) | 6.05a (0.10) | NS | 5.59a (0.06) | 5.45a (0.06) | 6.52a (0.06) | 6.21a (0.06) | NS |
| **Citrus aroma** | 4.31a (0.10) | 4.68a (0.10) | 4.54a (0.10) | 4.64a (0.10) | 4.49a (0.10) | 4.60a (0.10) | 4.58a (0.10) | 4.56a (0.10) | 4.51a (0.10) | NS | 4.22a (0.07) | 4.24a (0.07) | 4.86a (0.07) | 4.86a (0.07) | NS |
| **Grapefruit aroma** | 4.19a (0.10) | 4.39a (0.10) | 4.25a (0.10) | 4.20a (0.10) | 4.27a (0.10) | 4.22a (0.10) | 4.29a (0.10) | 4.38a (0.10) | 4.38a (0.10) | NS | 4.12a (0.06) | 4.00a (0.06) | 4.67a (0.06) | 4.30a (0.06) | NS |
| **Chemical aroma** | 3.91a (0.12) | 4.02a (0.12) | 4.04a (0.12) | 4.33a (0.12) | 3.98a (0.12) | 4.08a (0.12) | 3.94a (0.12) | 4.08a (0.12) | 4.16a (0.12) | NS | 3.91a (0.08) | 3.82a (0.08) | 4.26a (0.08) | 4.25a (0.08) | NS |
| **Deteriorated/rotten aroma** | 2.03a (0.08) | 2.02a (0.08) | 2.19a (0.08) | 2.06a (0.08) | 2.05a (0.08) | 2.01a (0.08) | 2.06a (0.08) | 2.04a (0.08) | 2.15a (0.08) | NS | 2.18a (0.05) | 2.01a (0.05) | 2.10a (0.05) | 1.99a (0.05) | NS |
| **Muddy/mouldy aroma** | 2.12a (0.06) | 2.13a (0.06) | 2.12a (0.06) | 2.23a (0.06) | 2.11a (0.06) | 2.06a (0.06) | 2.17a (0.06) | 2.16a (0.06) | 2.20a (0.06) | NS | 2.15a (0.04) | 2.02a (0.04) | 2.26a (0.04) | 2.15a (0.04) | NS |
| **Fruity aroma** | 3.89a (0.10) | 4.02a (0.10) | 3.75a (0.10) | 3.78a (0.10) | 3.87a (0.10) | 3.85a (0.10) | 3.82a (0.10) | 3.82a (0.10) | 3.90a (0.10) | NS | 3.79a (0.07) | 3.63a (0.07) | 4.07a (0.07) | 3.94a (0.07) | NS |

| | | | | | | | | | | | | | | | |
|---|---|---|---|---|---|---|---|---|---|---|---|---|---|---|---|
| **Green/grassy aroma** | 3.10a (0.08) | 3.22a (0.08) | 3.05a (0.08) | 3.15a (0.08) | 3.09a (0.08) | 3.02a (0.08) | 3.11a (0.08) | 3.24a (0.08) | 3.24a (0.08) | NS | 2.92a (0.06) | 2.90a (0.06) | 3.35a (0.06) | 3.37a (0.06) | NS |
| **Peely/peel oil aroma** | 3.58a (0.10) | 3.57a (0.10) | 3.45a (0.10) | 3.50a (0.10) | 3.52a (0.10) | 3.39a (0.10) | 3.39a (0.10) | 3.66a (0.10) | 3.54a (0.10) | NS | 3.25a (0.06) | 3.16a (0.06) | 3.81a (0.06) | 3.78a (0.06) | NS |
| **Soapy aroma** | 3.29a (0.11) | 3.46a (0.11) | 3.05a (0.11) | 3.39a (0.11) | 3.23a (0.11) | 2.99a (0.11) | 3.54a (0.11) | 3.31a (0.11) | 3.09a (0.11) | NS | 3.18a (0.07) | 3.14a (0.07) | 3.39a (0.07) | 3.32a (0.07) | NS |
| **Pungent aroma** | **2.96ab (0.10)** | **3.20a (0.10)** | **3.13a (0.10)** | **3.40a (0.10)** | **2.96b (0.10)** | **3.05ab (0.10)** | **3.15a (0.10)** | **3.23a (0.10)** | **3.14a (0.10)** | * | 2.87a (0.07) | 2.81a (0.07) | 3.45a (0.07) | 3.41a (0.07) | NS |
| **Woody/spicy aroma** | 2.44a (0.07) | 2.51a (0.07) | 2.34a (0.07) | 2.51a (0.07) | 2.43a (0.07) | 2.38a (0.07) | 2.49a (0.07) | 2.52a (0.07) | 2.40a (0.07) | NS | 2.39a (0.05) | 2.26a (0.05) | 2.60a (0.05) | 2.54a (0.05) | NS |
| **Sweet aroma** | 3.77a (0.10) | 3.78a (0.10) | 3.64a (0.10) | 3.72a (0.10) | 3.70a (0.10) | 3.66a (0.10) | 3.57a (0.10) | 3.68a (0.10) | 3.86a (0.10) | NS | 3.65a (0.07) | 3.46a (0.07) | 3.93a (0.07) | 3.79a (0.07) | NS |
| **Overall flavour intensity** | 6.02a (0.10) | 6.15a (0.10) | 6.24a (0.10) | 6.57a (0.10) | 6.37a (0.10) | 6.58a (0.10) | 6.63a (0.10) | 6.35a (0.10) | 6.35a (0.10) | NS | 6.38a (0.07) | 6.36a (0.07) | 6.41a (0.07) | 6.32a (0.07) | NS |
| **Sour flavour** | 5.77a (0.14) | 4.71a (0.14) | 4.23a (0.14) | 5.94a (0.14) | 5.14a (0.14) | 4.15a (0.14) | 6.09a (0.14) | 5.37a (0.14) | 4.76a (0.14) | NS | 5.20a (0.09) | 5.10a (0.09) | 4.99a (0.09) | 5.24a (0.09) | NS |
| **Sweet flavour** | 3.15a (0.11) | 4.78a (0.11) | 5.99a (0.11) | 3.02a (0.11) | 4.57a (0.11) | 5.95a (0.11) | 2.95a (0.11) | 4.16a (0.11) | 5.51a (0.11) | NS | 4.34a (0.07) | 4.43a (0.07) | 4.61a (0.07) | 4.44a (0.07) | NS |
| **Bitter flavour** | 4.64a (0.15) | 3.91a (0.15) | 3.27a (0.15) | 5.28a (0.15) | 4.20a (0.15) | 3.89a (0.15) | 5.81a (0.15) | 5.19a (0.15) | 4.50a (0.15) | NS | **4.91a (0.10)** | **4.64a (0.11)** | **4.21c (0.11)** | **4.33b (0.11)** | * |
| **Astringent flavour** | 5.10a (0.13) | 4.71a (0.13) | 4.05a (0.13) | 5.42a (0.13) | 4.85a (0.13) | 4.45a (0.13) | 5.53a (0.13) | 5.07a (0.13) | 4.75a (0.13) | NS | 5.04a (0.08) | 4.89a (0.08) | 4.85a (0.08) | 4.73a (0.08) | NS |
| **Citrus flavour** | 4.61a (0.10) | 4.45a (0.10) | 4.50a (0.10) | 4.29a (0.10) | 4.56a (0.10) | 4.47a (0.10) | 4.38a (0.10) | 4.33a (0.10) | 4.46a (0.10) | NS | 4.34a (0.07) | 4.27a (0.07) | 4.59a (0.07) | 4.60a (0.07) | NS |
| **Grapefruit flavour** | 4.24a (0.12) | 4.28a (0.12) | 4.04a (0.12) | 4.46a (0.12) | 4.51a (0.12) | 4.26a (0.12) | 4.87a (0.12) | 4.83a (0.12) | 4.48a (0.12) | NS | 4.55a (0.08) | 4.42a (0.08) | 4.50a (0.08) | 4.29a (0.08) | NS |
| **Bitter aftertaste** | 4.21a (0.15) | 3.67a (0.15) | 3.19a (0.15) | 5.10a (0.15) | 4.14a (0.15) | 3.54a (0.15) | 5.40a (0.15) | 4.93a (0.15) | 4.41a (0.15) | NS | 4.59a (0.10) | 4.40a (0.10) | 4.05a (0.10) | 4.12a (0.10) | NS |

[1] Mean values in a row for a specific two-way interaction effect printed in bold and with different letters are significantly different, * $p \leq 0.05$, ** $p \leq 0.01$, *** $p \leq 0.0001$; NS = not significantly different. Attribute intensity scale (0–10 cm) ranging from 'not intense' (0) to 'very intense' (10); [2]Aroma blend [Caryophylene, citral, nootkatone, aldehyde C8 (octanal), aldehyde C9 (nonanal), aldehyde C10 (decanal)] [3]Red color = 0.001 % solution (30 % Sunset yellow and 70 % Ponceau red); Yellow color = 0.0125% Quinoline yellow.

**Table S4.** Summary of sensory attribute mean values [1] [± standard error of means (SEM)] and significance of sweet x aroma and sweet x color two-way ANOVA interactions of the model grapefruit-like beverages as evaluated by a trained sensory panel (*n* = 16).

| Attributes | Sweet (°Brix) x Aroma mg/kg[2] | | | | | | | Sweet (°Brix) x Color[3] | | | | | | |
|---|---|---|---|---|---|---|---|---|---|---|---|---|---|---|
| | Sweet (Brix) | | | | | | | Sweet (°Brix) | | | | | | |
| | 8 Low | | 10 Medium | | 12 High | | | 8 Low | | 10 Medium | | 12 High | | |
| | Aroma mg/kg | | | | | | | Color | | | | | | |
| | 2.5 Low | 10 High | 2.5 Low | 10 High | 2.5 Low | 10 High | | Red | Yellow | Red | Yellow | Red | Yellow | |
| **Overall aroma intensity** | 5.57a[2] (0.08) | 6.36a (0.08) | 5.44a (0.08) | 6.38a (0.08) | 5.57a (0.08) | 6.35a (0.08) | NS | 6.11a (0.08) | 5.82a (0.08) | 5.94a (0.08) | 5.88a (0.08) | 6.13a (0.08) | 5.79a (0.08) | NS |
| **Citrus aroma** | 4.17a (0.08) | 4.84a (0.08) | 4.27a (0.08) | 4.88a (0.08) | 4.24a (0.08) | 4.85a (0.08) | NS | 4.60a (0.08) | 4.42a (0.08) | 4.52a (0.08) | 4.64a (0.08) | 4.50a (0.08) | 4.60a (0.08) | NS |
| **Grapefruit aroma** | 4.01a (0.08) | 4.44a (0.08) | 4.16a (0.08) | 4.53a (0.08) | 4.00a (0.08) | 4.50a (0.08) | NS | 4.43a (0.08) | 4.03a (0.08) | 4.46a (0.08) | 4.23a (0.08) | 4.30a (0.08) | 4.20a (0.08) | NS |
| **Chemical aroma** | 3.93a (0.10) | 4.20a (0.10) | 3.79a (0.10) | 4.26a (0.10) | 3.88a (0.10) | 4.30a (0.10) | NS | 4.15a (0.10) | 3.98a (0.10) | 3.99a (0.10) | 4.06a (0.10) | 4.12a (0.10) | 4.06a (0.10) | NS |
| **Deteriorated/rotten aroma** | 2.06a (0.06) | 2.04a (0.06) | 2.02a (0.06) | 2.06a (0.06) | 2.20a (0.06) | 2.04a (0.06) | NS | 2.16a (0.06) | 1.93a (0.06) | 2.10a (0.06) | 1.98a (0.06) | 2.16a (0.06) | 2.08a (0.06) | NS |
| **Muddy/mouldy aroma** | 2.14a (0.05) | 2.21a (0.05) | 2.05a (0.05) | 2.22a (0.05) | 2.08a (0.05) | 2.18a (0.05) | NS | 2.25a (0.05) | 2.10a (0.05) | 2.16a (0.05) | 2.10a (0.05) | 2.20a (0.05) | 2.06a (0.05) | NS |
| **Fruity aroma** | 3.66a (0.08) | 4.01a (0.08) | 3.72a (0.08) | 4.09a (0.08) | 3.75a (0.08) | 3.92a (0.08) | NS | 3.95a (0.08) | 3.71a (0.08) | 3.90a (0.08) | 3.91a (0.08) | 3.94a (0.08) | 3.73a (0.08) | NS |
| **Green/grassy aroma** | 2.87a (0.07) | 3.36a (0.07) | 2.94a (0.07) | 3.42a (0.07) | 2.91a (0,07) | 3.30a (0.07) | NS | 3.12a (0.07) | 3.11a (0.07) | 3.17a (0.07) | 3.19a (0.07) | 3.10a (0.07) | 3.10a (0.07) | NS |
| **Peely/peel oil aroma** | 3.13a (0.08) | 3.84a (0.08) | 3.30a (0.08) | 3.80a (0.08) | 3.18a (0.08) | 3.74a (0.08) | NS | 3.54a (0.08) | 3.43a (0.08) | 3.55a (0.08) | 3.55a (0.08) | 3.49a (0.08) | 3.42a (0.08) | NS |
| **Soapy aroma** | 3.35a (0.09) | 3.46a (0.09) | 3.18a (0.09) | 3.48a (0.09) | 2.96a (0.09) | 3.12a (0.09) | NS | 3.42a (0.09) | 3.39a (0.09) | 3.29a (0.09) | 3.37a (0.09) | 3.14a (0.09) | 2.94a (0.09) | NS |
| **Pungent aroma** | 2.92a (0.08) | 3.42a (0.08) | 2.78a (0.08) | 3.48a (0.08) | 2.83a (0.08) | 3.39a (0.08) | NS | 3.20a (0.08) | 3.14a (0.08) | 3.13a (0.08) | 3.13a (0.08) | 3.16a (0.08) | 3.06a (0.08) | NS |
| **Woody/spicy aroma** | 2.32a (0.06) | 2.64a (0.06) | 2.38a (0.06) | 2.59a (0.06) | 2.27a (0.06) | 2.47a (0.06) | NS | 2.53a (0.06) | 2.44a (0.06) | 2.52a (0.06) | 2.45a (0.06) | 2.43a (0.06) | 2.31a (0.06) | NS |
| **Sweet aroma** | 3.44a (0.09) | 3.93a (0.09) | 3.58a (0.09) | 3.86a (0.09) | 3.64a (0.09) | 3.79a (0.09) | NS | 3.74a (0.09) | 3.63a (0.09) | 3.76a (0.09) | 3.68a 0.09) | 3.88a (0.09) | 3.56a (0.09) | NS |
| **Overall flavour intensity** | 6.46a (0.08) | 6.36a (0.08) | 6.31a (0.08) | 6.28a (0.08) | 6.35a (0.08) | 6.46a (0.08) | NS | 6.51a (0.08) | 6.31a (0.08) | 6.29a (0.08) | 6.30a (0.08) | 6.39a (0.08) | 6.42a (0.08) | NS |
| **Sour flavour** | 5.96a (0.11) | 5.91a (0.11) | 5.07a (0.11) | 5.08a (0.11) | 4.40a (0.11) | 4.35a (0.11) | NS | 6.01a (0.11) | 5.86a (0.11) | 4.96a (0.11) | 5.19a (0.11) | 4.31a (0.11) | 4.44a (0.11) | NS |
| **Sweet flavour** | 2.92a (0.09) | 3.16a (0.09) | 4.38a (0.09) | 4.63a (0.09) | 5.85a (0.09) | 5.78a (0.09) | NS | 3.09a (0.09) | 2.99a (0.09) | 4.49a (0.09) | 4.52a (0.09) | 5.85a (0.09) | 5.78a (0.09) | NS |

| | | | | | | | | | | | | | | |
|---|---|---|---|---|---|---|---|---|---|---|---|---|---|---|
| **Bitter flavour** | 5.51a (0.12) | 4.98a (0.12) | 4.80a (0.12) | 4.07a (0.12) | 4.02a (0.12) | 3.76a (0.12) | NS | 5.30a (0.12) | 5.18a (0.12) | 4.55a (0.12) | 4.32a (0.12) | 3.82a (0.12) | 3.96a (0.12) | NS |
| **Astringent flavour** | 5.42a (0.10) | 5.28a (0.10) | 4.96a (0.10) | 4.79a (0.10) | 4.52a (0.10) | 4.31a (0.12) | NS | **5.62a (0.10)** | **5.08b (0.10)** | **4.92b (0.10)** | **4.83b (0.10)** | **4.30c (0.10)** | **4.52c (0.10)** | * |
| **Citrus flavour** | 4.29a (0.08) | 4.56a (0.08) | 4.33a (0.08) | 4.56a (0.08) | 4.29a (0.08) | 4.67a 0.08) | NS | **4.59a (0.08)** | **4.26b (0.08)** | **4.40a (0.08)** | **4.50a (0.08)** | **4.42a (0.08)** | **4.54a (0.08)** | * |
| **Grapefruit flavour** | 4.45a (0.09) | 4.60a (0.09) | 4.69a (0.09) | 4.38a (0.09) | 4.32a (0.09) | 4.20a (0.09) | NS | 4.60a (0.09) | 4.45a (0.09) | 4.60a (0.09) | 4.48a (0.09) | 4.39a (0.09) | 4.14a (0.09) | NS |
| **Bitter aftertaste** | 5.03a (0.12) | 4.77a (0.12) | 4.54a (0.12) | 3.96a (0.12) | 3.91a (0.12) | 3.52a (0.12) | NS | 4.94a (0.12) | 4.87a (0.12) | 4.36a (0.12) | 4.13a (0.12) | 3.65a (0.12) | 3.77a (0.12) | NS |

[1] Mean values in a row for a specific two-way interaction effect printed in bold and with different letters are significantly different;, * $p \leq 0.05$, ** $p \leq 0.01$, *** $p \leq 0.0001$; NS = not significantly different Attribute intensity scale (0-10 cm) ranging from 'not intense' (0) to 'very intense' (10); [2] Aroma blend [Caryophylene, citral, nootkatone, aldehyde C8 (octanal), aldehyde C9 (nonanal), aldehyde C10 (decanal)] [3] Red color = 0.001 % solution (30 % Sunset yellow and 70 % Ponceau red); Yellow color = 0.0125% Quinoline yellow.
